# The Epidemiology of Hepatitis C Virus in the Maghreb Region: Systematic Review and Meta-Analyses

**DOI:** 10.1371/journal.pone.0121873

**Published:** 2015-03-24

**Authors:** Fatima A. Fadlalla, Yousra A. Mohamoud, Ghina R. Mumtaz, Laith J. Abu-Raddad

**Affiliations:** 1 Infectious Disease Epidemiology Group, Weill Cornell Medical College, Qatar, Cornell University, Qatar Foundation, Education City, Doha, Qatar; 2 College of Medicine, University of Toledo, Toledo, Ohio, United States of America; 3 Department of Healthcare Policy and Research, Weill Cornell Medical College, Cornell University, New York, New York, United States of America; 4 Vaccine and Infectious Disease Division, Fred Hutchinson Cancer Research Center, Seattle, Washington, United States of America; UCL Institute of Child Health, University College London, UNITED KINGDOM

## Abstract

**Objective:**

To systematically review and synthesize available epidemiological data on hepatitis C virus (HCV) prevalence and incidence in the Maghreb region and to estimate the country-specific population-level HCV prevalence.

**Methods:**

We conducted a systematic review of HCV antibody prevalence and incidence in the Maghreb countries as outlined by the PRISMA guidelines. Meta-analyses were conducted using DerSimonian-Laird random-effect models with inverse variance weighting to pool HCV prevalence estimates among general population groups.

**Results:**

We identified 133 HCV prevalence measures and two HCV incidence measures. Among high risk groups, HCV prevalence ranged between 22% and 94% among people who inject drugs, 20% and 76% among dialysis patients, and 2% and 51% among hemophiliacs. Among intermediate-risk groups, considerable but widely variable HCV prevalence was found. Most common risk factors cited across studies were the duration of dialysis, number of transfusions, and having a history of surgery or dental work. The national HCV prevalence in Algeria was estimated at 0.3% (95%CI: 0.1–0.5), Libya 1.2% (95%CI: 1.1–1.3), Mauritania 1.1% (95%CI: 0–2.3), Morocco 0.8% (95%CI: 0.5–1.2), and Tunisia 0.6% (95%CI: 0.5–0.8).

**Conclusions:**

HCV prevalence in the Maghreb region of the Middle East and North Africa is comparable to that in developed countries of about 1%. HCV exposures appear often to be linked to medical care and are suggestive of ongoing transmission in such settings. Injecting drug use appears also to be a major, though not dominant, contributor to HCV transmission. Further research is needed to draw a more thorough understanding of HCV epidemiology, especially in the countries with limited number of studies. HCV prevention policy and programming in these countries should focus on the settings of exposure.

## Introduction

The Middle East and North Africa (MENA) region appears to have the highest prevalence of hepatitis C virus (HCV) worldwide [1, 2]. The highest national prevalence of HCV globally is found in Egypt (14.7%) [3, 4]. Questions remain as to the prevalence of HCV in the rest of North Africa and to what extent, if any, are this region’s rates influenced by the large HCV reservoir in Egypt.

The Maghreb region of North Africa borders Egypt to the east. Geographically, the region encompasses Algeria, Libya, Mauritania, Morocco and Tunisia. The combined population of this region is approximately 88 million people [5], or over one-fifth of the population of MENA. Our objective in this study was to systematically review and synthesize all epidemiological data on HCV antibody prevalence and incidence among the different population groups in the Maghreb; and to estimate the national population-level HCV prevalence for each of its five countries. The study is conducted under the umbrella of the MENA HCV Synthesis Project; an ongoing effort to characterize HCV epidemiology in the MENA region. The ultimate goal of this project is to provide the empirical evidence necessary for policy makers and public health stakeholders to set the key research, policy, and programming priorities for the MENA region.

## Methods

### Data Sources and Search Strategy

We conducted a systematic review of the literature following the Preferred Reporting Items for Systematic Reviews and Meta-analyses (PRISMA) guidelines (S1 Table) [6]. The primary outcome measure of interest was HCV antibody prevalence (sero-prevalence) and incidence (sero-incidence) in the Maghreb region. A secondary outcome measure of interest was HCV RNA prevalence among HCV antibody positive patients.

Main data sources for this review were PubMed (Medline) and Embase databases. No language restrictions were imposed in either database. While our PubMed search had no year limit, we restricted the search in Embase to articles published after 1980. However, since HCV was discovered in 1989, no effect of this restriction on our results is expected. The search criteria utilized *MeSH/Emtree* terms in PubMed and Embase, respectively, and text terms. Full details of the search criteria are presented in S1 Box. We used broad search criteria to insure completeness. Additional sources of data were primarily obtained through the MENA HIV/AIDS Epidemiology Synthesis Project database [7, 8]. These included international organizations’ reports and country-level reports.

### Study Selection

All records found through PubMed and Embase were initially screened to remove duplicates. The title and abstract for the remaining records were screened by two investigators (FAF and YAM) for relevance. Any uncertainty was settled through consultation with members of the study team. All relevant and potentially relevant full-text articles on the sero-prevalence or sero-incidence of HCV in the Maghreb were identified and subsequently screened. Case series, case reports, and reviews were excluded. We only included studies with primary data in our analysis. Publications reporting the same data were considered duplicates and counted as one publication. The bibliographies of relevant reviews were compared to our records, and studies reporting prevalence or incidence, not initially identified or retrieved, were added to our study.

### Data Extraction

Data from relevant records were abstracted by two investigators (FAF and YAM) for the following indicators: author, year of publication, year of study, country of study, study site, study design, sampling technique, population, sample size, sero-prevalence, RNA prevalence, incidence, and risk factors for HCV infection. Any disagreements were discussed and settled among the study team. Articles in French were abstracted by one author (GRM). Articles in Arabic were abstracted by two of the authors (FAF) and (LJA). Data was extracted from abstracts when the full-text was not available. Though articles identifying HCV risk factors were not specifically searched for, risk factor data was abstracted from relevant reports when available.

### Data Synthesis

Studies were organized by country and by study population. Study population was divided into categories according to the perceived risk of acquiring HCV infection. Four categories were defined: 1) high risk groups consisting of people who inject drugs (PWID), dialysis patients, hemophiliacs, and multi-transfused patients, among others; 2) intermediate risk groups including diabetics, barbers and others potentially exposed to HCV at intermediate risk such as healthcare workers; 3) low risk groups representing the general population including blood donors, pregnant women, healthy adults, army recruits, and healthy controls from case-control studies, among others; and 4) special clinical populations including renal transplant patients, hepatocellular carcinoma (HCC) patients, liver disease patients, and patients with psoriasis, among others. This latter category represents patients with clinical conditions associated with HCV infection or patients with specific diseases that require clinical care, and thus can be exposed to HCV in medical care facilities. These populations were grouped together as special clinical populations since the level of exposure to HCV is uncertain, and accordingly, it is difficult to categorize them among any of the other three population groups.

### Quantitative Analysis

Pooled estimates for HCV prevalence among the general population were calculated for Algeria, Morocco and Tunisia since we had a sufficient number of studies to power a meta-analysis in each of these countries. The analysis was conducted using Stata/SEv13 and R2.15.3. Estimates were pooled using a DerSimonian-Laird random effects model which assumes that the true effect size could vary from study to study, and that the true effects are normally distributed [9]. The model therefore accounts for both sampling variation and heterogeneity in effect size. Individual studies’ effect sizes were weighted using their inverse variance. The variance of the raw proportions was stabilized by transforming the proportions using the Freeman-Tukey type double-arcsine square-root transformation [10]. The back-transformed pooled proportions were then calculated using Miller’s inverse transformation with the harmonic mean of the sample sizes [11]. To examine the magnitude of the variation between studies due to heterogeneity rather than chance, we quantified the heterogeneity using the I^2^ measure and its confidence interval [12]. We considered a two-sided probability value <0.10 as significant.

A meta-analysis was not performed for each of Libya and Mauritania. While Libya presented various studies estimating the prevalence of HCV in the general population; the best estimate was chosen as that reported through the Libyan National Survey [13] due to its nationally representative sampling technique and large sample size (n = 65,711). Only one study was identified in Mauritania and therefore a meta-analysis was not possible.

## Results

### Search Results

**Fig 1:**
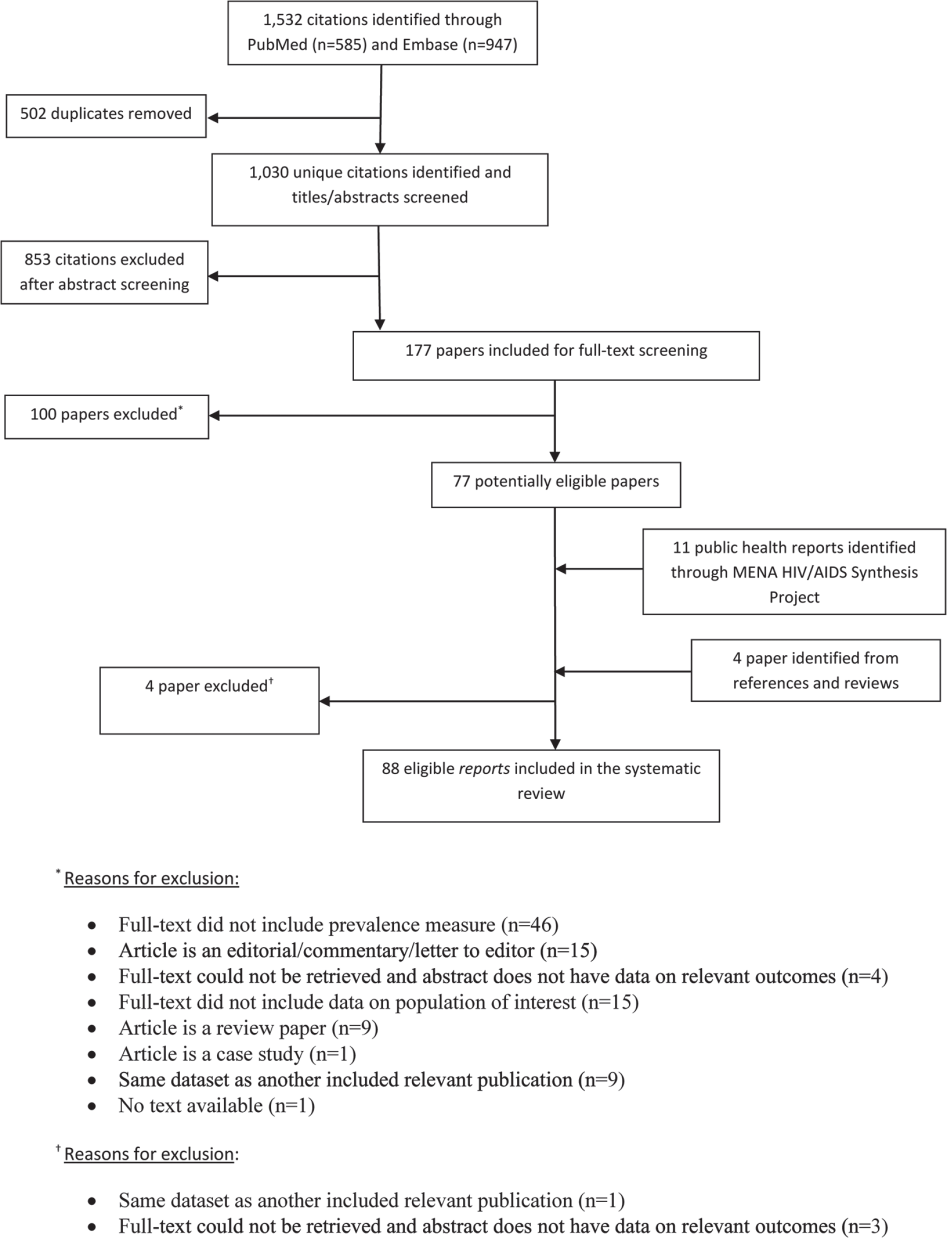
Flow chart of article selection adapted from the PRISMA 2009 guidelines [6].

A schematic diagram of the selection process is outlined in Fig. 1, adapted from the PRISMA 2009 flow diagram [6]. We identified a total of 1,532 records (585 through PubMed and 947 through Embase) as of December 11, 2013. Of these records, 502 duplicates were identified and excluded. Screening of the remaining titles and abstracts yielded 177 potentially relevant articles. Full-text records of those were retrieved and screened with the exception of four articles for which the full-text could not be retrieved and the abstracts did not report the relevant outcome. Screening of full-texts identified 77 articles for inclusion in the review. Four additional records were identified through screening of bibliographies of reviews; however, they were subsequently excluded as their full-texts could not be retrieved or they were a duplicate for a study already included. An additional 11 relevant records were included through the MENA HIV/AIDS Synthesis Project database (one international organization report and 10 country-level reports).

### HCV Prevalence Overview

Data on HCV prevalence as well as other indicators were abstracted from the 88 relevant records, and presented in Table 1. In total, 133 HCV prevalence measures were identified by our study. Most reports presenting HCV prevalence in the Maghreb were from Morocco (n = 38) and Tunisia (n = 60). Populations studied to estimate HCV prevalence ranged from general population groups such as blood donors, pregnant women, and healthy adults, to high risk groups such as PWID, dialysis patients, hemophiliacs, and multi-transfused patients. The following is a synthesis of HCV prevalence in each of the Maghreb countries:

**Table 1:** Studies reporting hepatitis C virus (HCV) prevalence in the Maghreb countries.

Citation	Year of Data Collection	Study Site	Study Design	Population	Sample Size	Antibody prevalence	RNA Prevalence among Anti-HCV Positive
**ALGERIA**							
**High Risk**							
Saidane,11 [14]	2010–2011	Hospital	CS	Hemophiliacs	64	30%	
**General Population**							
Aidaoui,08 [15]	2008	Hospital	CS	Pregnant women	3044	0.63%	
Ayed, 95 [16]	1992–1993	Hospital		Pregnant women	715	0.19%	
Ayed, 95 [16]	1992–1993	Blood transfusion center	CS	Blood donors	1112	0.18%	
**LIBYA**							
**High Risk**							
Mirzoyan,13 [17]	2010		CS	People who inject drugs	328	94.2%	
Yerly,01 [18]	1998–1999	Hospital	CS	HIV patients (children) suspected of acquiring infection paranterally	111	46%	
Yerly,01 [18]	1998–1999	Hospital	CS	HIV patients (children) suspected of acquiring infection paranterally	37	43%	
Elzouki,93 [19]		Hospital	CS	Dialysis patients	47	42.5%	
Alashek,12 [20]	2009–2010	Hemodialysis centers	CS	Dialysis patients	2382	32.3%	
Elzouki,95 [21]		Hospital	CS	Dialysis patients	153	21%	72%
Daw,02 [22]	1999–2001		CS	Dialysis patients	200	20.5%	
Daw,02 [22]	1999–2001			Multi-transfused patients	250	10.8%	
**Intermediate Risk**							
Yerly,01 [18]	1998–1999	Hospital	CS	Parents of children with HIV suspected of acquiring infection paranterally	46	4.35%	
Kutrani,07 [23]	2003	Hospital	CS	Non-Libyan patients referred to the infectious disease department	281	54.09%	
Kutrani,07 [23]	2003	Hospital	CS	Libyan patients referred to the infectious disease department	1019	44.95%	
Alashek,10 [24]	2009	Dialysis units	CS	Diabetic patients	749	24.4%	
Ziglam,12 [25]	2006	Prisons	CS	Prisoners	6371	23.7%	
Valadez,13 [26]	2010–2011	Community probability based sample	CS	Male sex workers	227	7.3%	
Saleh,94 [27]	1992	Hospital	CS	Hospital workers	190	6.8%	69.23%
Valadez,13 [26]	2010–2011	Community probability based sample	CS	Female sex workers	69	5.2%	
Abddulsalam Bagar,10 [28]	2007–2008	Hospital	CS (Retrospective)	Patients with unspecified disease	17419	3.67%	
Franka,09 [29]	2004	Hospital	CS	Medical waste handlers	300	2.7%	
Daw,02 [22]	1999–2001		CS	Hospital workers	459	2.0%	
Franka,09 [29]	2004	Medical management facility	CS	Non-medical waste handlers	300	0%	
**General Population**							
Shabash,10 [30]				General population	878	23.2%	
Saleh,94 [27]	1992	Hospital	CS	Blood donors	76	6.6%	60%
Shabash,10 [31]	2003–2008	Laboratory	CS (Retrospective)	General population	1008214	1.77%	
Daw,02 [22]	1999–2001		CS	Healthy adults	800	1.6%	
Daw,02 [22]	1999–2001		CS	Blood donors	1200	1.2%	
Daw,14 [13]	2008	Libyan national survey	CS	General population	65711	1.19%	
Elzouki,95 [32]		Blood Bank	CS	Blood donors	86	0.9%	
**Special Clinical Population**							
Elbouaishi,10 [33]		Hospital	CS (Retrospective)	Nephrotic syndrome patients (children)	329	8.51%	
**MAURITANIA**							
**General Population**							
Lo,99 [34]	1998	Hospital	CS	Blood donors	349	1.1%	
**MOROCCO**							
**High Risk**							
HIV Integrated Behavioral and Biological Surveillance Survey,12 [35]	2011–2012		CS	People who inject drugs	274	79.2%	
Boulaajaj,05 [36]	1983–2002	Hospital	CS (Retrospective)	Dialysis patients	126	76%	
Sekkat,08 [37]	2003–2004	Dialysis units	CS	Dialysis Patients	303	68.3%	
Amar,05 [38]		Dialysis units	CS	Dialysis patients	85	54.12%	
HIV Integrated Behavioral and Biological Surveillance Survey,12 [35]	2010–2011		CS	People who inject drugs	261	45.6%	
Benjelloun,96 [39]		Hemophilia treatment center	CS	Hemophiliacs	118	42.4%	
Bousfiha,99 [40]	1999	Hemophilia treatment center	CS	Hemophiliacs (children)	39	41%	
Benjelloun,96 [39]		Dialysis units	CS	Dialysis patients	114	35.1%	
HIV Integrated Behavioral and Biological Surveillance Survey,12 [35]	2010–2011		CS	People who inject drugs	22	31.8%	
HIV Integrated Behavioral and Biological Surveillance Survey,12 [35]	2011–2012		CS	People who inject drugs	83	22.9%	
El Khorassani,10 [41]	1981–2006	Hospital	CS (Retrospective)	Hemophiliacs	262	2.29%	
**Intermediate Risk**							
Benjelloun,96[39]		STD center	CS	HIV patients	116	19.8%	
Cacoub,00 [42]	1995–1996	Hospital	CS	Inpatients	280	10.36%	75%
Cacoub,00 [42]	1995–1996	Hospital	CS	Outpatients	503	6.16%	
Rebbani,13 [43]	2006–2010	Infectious disease center	CS	HIV patients	503	5.4%	
Zahraoui-Mehadji,04 [44]	2001		CS	Traditional barbers	150	5%	
Benjelloun,96[39]		STD center	CS	STD patients	2088	3%	
Belbacha,11 [45]	2007		CS	Traditional barbers	267	1.10%	
Lahlou Amine,10 [46]	2005–2006	Hospital	CS (Retrospective)	Inpatients	2350	0.76%	
**General Population**							
Benouda,09 [47]	2005–2007	Laboratory	CS	General population	8326	1.93%	
Baha,13 [48]	2005–2011	Nationwide	CS	General population	41269	1.58%	
Belbacha,11 [45]	2007			Clients of barbers	529	1.30%	
Benani,11 [49]			CS	General population	24646	1.10%	
Benjelloun,96 [39]		Blood transfusion center	CS	Blood donors	1000	1.1%	
Benjelloun,96 [39]		Hospital	CS	Pregnant women	676	1.0%	
Aqodad,11 [50]	2006–2007	Blood bank	CS	Blood donors	777	0.8%	
Baha,13 [48]	2005–2011	Blood transfusion center	CS	Blood donors	169605	0.62%	
Regional Database on HIV/AIDS. WHO Regional Office for the Eastern Mediterranean,11 [51]			CS	ANC and family planning clinic attendees		0.5%	
Lahlou Amine,11 [46]	2005–2006	Hospital	CS (Retrospective)	Army recruits	16000	0.35%	
Zohoun,11 [52]	2008–2009	Hospital	CS (Retrospective)	Blood donors	19801	0.2%	
Regional Database on HIV/AIDS WHO Regional Office for the Eastern Mediterranean,11 [53]	2010		CS	Blood donors	132197	0.16%	
**Special Clinical Population**							
Rioche,91 [54]	1983–1986	Dialysis units	CS	Non-A and Non-B chronic hepatitis patients	38	73.7%	
Ezzikouri,09 [55]	2003–2006	Hospital	CC	Hepatocellular carcinoma patients	96	57.3%	
Rioche,91 [54]	1983–1986	Hospital	CS	Non-A and Non-B acute hepatitis patients	90	44.4%	
Lakhoua Gorgi,10 [56]	1987–2004	Hospital	CS	Renal transplant patients	57	19.3%	
Radoui,10 [57]	1998–2008	Hospital	CS	Renal transplant patients	69	10.1%	
Mohammed,12 [58]	2005–2011	Hospital	CS (Retrospective)	Hepatic steatosis patients	79	3.8%	
Lahlou Amine,10 [46]	2005–2006	Hospital	CS (Retrospective)	Patients consulting for HCV infection	7050	2.32%	
Bousfiha,99 [59]	1999	Hospital	CS	Acute hepatitis patients (children)	130	0%	
**TUNISIA**							
**High Risk**							
Ayed,03 [60]	2001	Dialysis units/Overall	CS	Dialysis patients	1394	22.24%	76.7%
Belarbi,13 [61]	2012	Outpatient hospital	CS (Retrospective)	People who inject drugs	23	21.7%	
Ayed,03 [60]	2001	Dialysis units/Central			1314	18.47%	60.5%
Ayed,03 [60]	2001	Dialysis units/Northwest			358	15.36%	70.9%
Ayed,03 [60]	2001	Dialysis units/Northern			279	15.7%	90.9%
Ayed,03 [60]	2001	Dialysis units/Southern			796	14.57%	71.5%
Bejaoui,13 [62]		Medical institutions	CS (Retrospective)	Thalassemia patients	391	6.1%	
Hannachi,11 [63]	2008–2009	Hospital	CC	Multi-transfused patients	107	4.7%	
Tunisia Ministry of Health,10 [64]	2009		CS	People who inject drugs	715	29.1%	
Djebbi,08 [65]	2003	Hospital	CS	Hemophiliacs	95	50.5%	87.5%
Sassi,00 [66]		Dialysis units	CS	Dialysis patients	58	46.5%	51%
Hmida,95 [67]		Dialysis units	CS	Dialysis patients	235	45.1%	
Ben Othman,04 [68]	2000–2002	Dialysis units	CS	Dialysis patients	276	32.6%	78.89%
Ayed,03 [60]	2001	Dialysis units/Northeastern			199	30.15%	93.33%
Hachicha,95 [69]		Dialysis units	CS	Dialysis patients	235	42%	
Hmaied,06 [70]	2001–2003	Dialysis units	CS	Dialysis patients	395	20%	73%
Jemni,94 [71]		Dialysis units	CS	Dialysis patients	63	42%	
Langar,05 [72]		Hospital	CS	Hemophiliacs	70	50%	
**Intermediate Risk**							
Kilani,07 [73]	1997–2005	Hospital	CS	HIV patients	362	39.7%	
Maaref,11 [74]	2006	Hospital	CS	HIV patients	125	26.4%	
Larabi,01 [75]	1997–1999	Hospital	CS	Inpatients	542	20.3%	
Kaabia,09 [76]	2003	Hospital	CS	Diabetic patients	1269	1.3%	
Znazen,10 [77]	2007	Hospital	CS	Female sex workers	188	1.1%	
Kaabia,09 [78]	2005	Hospital	CS	Hospital workers	885	1%	
**General Population**							
Coursaget,90 [79]	1982–1986		CS	Blood donors	99	3.0%	
Coursaget,95 [80]		Hospital	CS	Blood donors	45	2.2%	
Krichen,01 [81]	1995–1997		CS	Blood donors	42623	1.71%	
Mejri,05 [82]	1996	Households	CS	General population	4157	1.7%	82%
Larabi,01 [75]	1997–1999	Hospital	CS	Blood donors	3480	1.18%	
Slama,91 [83]		Secondary schools	CS	Blood donors	2006	1.09%	
HIV/AIDS Quarterly Report,07 [84]	2007- 2^nd^ Quarter		CS		24247	0.87%	
Gorgi,98 [85]	1994–1996	Community	CS	General population	3079	0.71%	
HIV/AIDS Quarterly Report,07 [84]	2007- 3^rd^ Quarter		CS		10526	0.64%	
Hannachi,11 [63]	2008–2009	Hospital	CC	Controls of a case-control study	160	0.6%	
Kaabia,09 [76]	2003	Hospital	CS	Controls of a case-control study	1315	0.6%	
Hatira,00 [86]	1994–1997	Blood bank	CS	Blood donors	34130	0.56%	
HIV/AIDS Quarterly Report,06 [87]	2006- 3^rd^ Quarter		CS		20803	0.54%	
Triki,94 [88]		Laboratory	CS	Healthy adults	735	0.40%	
Triki,97 [89]	1987–1994		CS	Blood donors	785	0.4%	
HIV/AIDS Quarterly Report,07 [84]	2007- 4^th^ Quarter		CS		58368	0.36%	
HIV/AIDS Quarterly Report,06 [87]	2006- 2^nd^ Quarter		CS	Blood donors	31115	0.34%	
Kallel,11 [90]			CC	Controls of a case-control study	300	0.33%	
HIV/AIDS Quarterly Report,06 [87]	2006- 4^th^ Quarter		CS		28428	0.30%	
HIV/AIDS Quarterly Report,07 [84]	2007- 1^st^ Quarter		CS		259890	0.26%	
Hannachi,11 [91]	2006	Hospital	CS	Pregnant women	404	0.2%	
Mejri,05 [82]	1996	Households	CS	General population	7350	0.2%	71%
Abid,97 [92]	1994	Blood bank	CS	Blood donors	43000	0.18%	
Mahjoub,13 [93]	2000–2010		CS	Blood donors (military)	182996	0.14%	
Samoud,11 [94]			CC	Healthy adults	64	0%	
**Special Clinical Populations**							
Bouzgarrou,11 [95]	2005–2008	Hospital	CS	Chronic hepatitis patients	77	80.52%	
Coursaget,90[79]	1982–1986		CS	Non-A and Non-B acute hepatitis patients	25	48.0%	
Triki,94 [88]		Laboratory	CS	Cirrhosis patients	168	43%	
Coursaget,92 [96]			CS	Cirrhosis patients	23	31%	
Lakhoua Gorgi,10 [56]	1987–2004	Hospital	CS	Renal transplant patients	115	20.9%	91.7%
Triki,94 [88]		Laboratory	CS	Hepatocellular carcinoma patients	31	19%	
Coursaget,95 [80]		Hospital	CS	Acute hepatitis patients	45	18%	
Coursaget,90[79]	1982–1986		CS	HBV patients	28	14.3%	
Samoud,11 [94]			CC	Psoriatic patients	41	9.75%	
Coursaget,92 [96]			CS	Acute hepatitis patients	25	8%	
Hannachi,10 [97]	2010		CS	HBV patients	273	3.8%	
Kallel,11 [90]			CC	Inflammatory bowel disease patients	150	2%	
**ALGERIA, MOROCCO and TUNISIA**							
**General Population**							
Bahri,11 [98]	2002–2005	Multi-center study	CC	Controls of a case-control study	250	4.40%	

CS: cross-sectional study design, CC: case-control study design, STD: sexually transmitted disease, HBV: hepatitis B virus

Note: Citations are sorted within each risk group in descending order of prevalence.

#### Algeria

The single study from Algeria reporting on a high risk group examined hemophiliacs, reporting a prevalence of 30% [14] (Table 1). Three studies reported general population HCV prevalence estimates in Algeria. Prevalence of 0.6% [15] and 0.2% [16] was reported among pregnant women, and HCV prevalence of 0.2% [16] was reported among blood donors.

#### Libya

Among high risk groups in Libya, PWID were reported to have the highest HCV prevalence at 94% [17] (Table 1). Dialysis patients had a prevalence ranging from 21% [22] to 43% [19]. One study examined the prevalence among children infected with HIV and children with HIV who were referred to a specific hospital (both children groups were suspected to have had parenteral exposure to HIV) [18]. HCV prevalence was 46% and 43%, respectively [18]. Among the parents of the children with HIV who were referred to this specific hospital, HCV prevalence was 4% [18].

HCV was measured among several populations at intermediate risk including diabetics (24%) [24], prisoners (24%) [25], male sex workers (7%) [26], female sex workers (5%) [26], hospital care workers (2%) [22], and medical (3%) and non-medical (0%) waste handlers [29] (Table 1). Among patients referred to an infectious disease department, with no further specification, persons of non-Libyan backgrounds had an HCV prevalence of 54% while their Libyan counterparts had a prevalence of 45% [23].

Five reports and one national survey reported HCV prevalence among the general population in Libya [13, 22, 27, 30–32]. Estimates hovered around 1–2% with two outliers of much larger prevalence (Table 1).

Only one study was conducted among special clinical populations in Libya (Table 1). The study reported HCV prevalence of 9% among children with nephrotic syndrome [33].

#### Mauritania

Only one study measured HCV prevalence in Mauritania. The study tested 349 blood donors at a national hospital, reporting a prevalence of 1% [34]. The majority of donors were males (333 vs. 16).

#### Morocco

Among high risk groups, HCV prevalence was measured among PWID, but through small samples, at 32% in Nador and 23% in Tanger [35] (Table 1). However, more recently, it was measured among PWID in the same cities, using large probability-based samples (respondent-driven sampling), to be 79% in Nador and 46% in Tanger [35]. Among dialysis patients, HCV prevalence ranged from 35% [39] to as high as 76% [36]. Hemophiliacs had variable rates as reported from three studies with one examining rates in children. At 42%, the earliest of the three studies reported the highest HCV prevalence among hemophiliacs in Morocco [39]. The second study, conducted among children, reported a comparable prevalence of 41% [40]. The third, and most recent study, reported the lowest HCV prevalence among hemophiliacs at 2% [41].

Benjelloun *et al*. examined HCV prevalence among two intermediate risk populations including those having a history of a sexually transmitted disease (STD) and those infected with HIV (likely through a sexual mode of transmission) [39]. For those with a history of STD, HCV prevalence was 3%, while for those who tested positive for HIV, HCV prevalence was 20% [39]. Another study of HIV infected patients reported an HCV prevalence of 5% [43]. Among hospital attendees, an early study reported HCV prevalence of 10% among inpatients and 6% among outpatients [42]. A more recent study reported an HCV prevalence of 0.8% among inpatients [46]. Traditional barbers were tested for HCV in two studies in Morocco where the prevalence was reported to be 1% [45] and 5% [44].

Estimates of HCV prevalence in the general population in Morocco came from eight reports including 11 prevalence measures on pregnant women, blood donors, and army recruits, among others. HCV prevalence ranged between 0.2% and 2% (Table 1).

Among special clinical populations, two studies examined HCV prevalence among those who had a renal transplant reporting estimates of 19% [56] and 10% [57]. HCV prevalence among HCC patients was 57% [55]. Individuals with chronic or acute hepatitis had prevalence estimates of 74% and 44% [54].

#### Tunisia

Among high risk groups, HCV prevalence was measured among PWID to be 22% (small sample) [61] and 29% [64] (Table 1). Among dialysis patients, several studies reported different measures in different areas which ranged between 15% [60] and 47% [66]. Djebbi *et al*. and Langar *et al*. measured HCV prevalence among hemophiliacs and reported similar results of 51% [65] and 50% [72], respectively. HCV prevalence among multi-tranfused patients was 5% [63], while it was 6% among thalassemia patients [62].

Among intermediate risk populations, HCV prevalence was measured to be 1% among diabetics [76], 20% among inpatients and referred patients (non-specified) [75], and 1% among hospital employees [78]. Among individuals testing positive for HIV, HCV prevalence was 26% [74] and 40% [73]. Among female sex workers, it was 1% [77].

Of the Maghreb countries, Tunisia had the largest number of studies measuring HCV prevalence among general population groups. Many of the studies had large samples (typically over 1,000), and were among populations such as blood donors, pregnant women, and healthy adults, among others. In total, 15 articles and seven country-level reports provided measures for Tunisia’s HCV prevalence in the population at large (24 prevalence measures). HCV prevalence in the general population abstracted from these studies ranged between 0% and 3% (Table 1).

Notably, relatively high HCV prevalence was reported among special clinical populations. HCV prevalence ranged between 31% [96] and 43% [88] among cirrhosis patients, and was as high as 21% [56] among renal transplant recipients, and as high as 19% [88] among HCC patients. Two studies examining chronic and Non-A and Non-B acute hepatitis reported high HCV prevalence at 81% [95] and 48% [79], respectively. Two other studies among individuals with acute viral hepatitis estimated HCV prevalence at 8% [96] and 18% [80].

### HCV Incidence Overview

Only two studies, both in dialysis units, reported empirical measures of HCV incidence in the Maghreb. The first in Tunisia, by Ben Othman *et al*., reported an HCV incidence rate of 2.8 per 100 person-years in 2000–2002 [68]. The second study in Morocco, by Sekkat *et al*., estimated HCV incidence rate at 9.7 per 100 person-years in 2003–2004 [37].

### Risk Factors

Risk factors were most typically identified in studies conducted among high risk or intermediate risk populations. These included dialysis patients, inpatients, and patients referred to an infectious disease department. Common risk factors identified across countries were the duration of dialysis [21, 38, 66], history and number of transfusions [38, 66], and whether individuals had a history of surgery or dental work [23, 42].

### HCV Genotypes

Genotype 1 is the most common HCV genotype consistently reported for most countries in the Maghreb [99, 100]. According to a study by Messina *et al*., HCV genotype 1 accounts for 82% of HCV infections in Algeria, 44% of infections in Libya, 74% of infections in Morocco, and 41% of infections in Tunisia [99]. Another study by Ezzikouri *et al*. places genotype 4 as the most common genotype in Libya, and genotype 1 as the second most common (genotype 4: 36% vs. genotype 1: 33%) [100]. For Algeria, Morocco, and Tunisia, genotype 2 is consistently reported as the second most common genotype [99, 100]. The genotype distribution diversity, as measured by the Shannon Diversity Index, shows high diversity in Libya and Tunisia and low diversity in Algeria and Morocco [99]. No data seems to be available for HCV genotype distribution in Mauritania.

### National Population-Level HCV Prevalence Estimates

**Fig 2:**
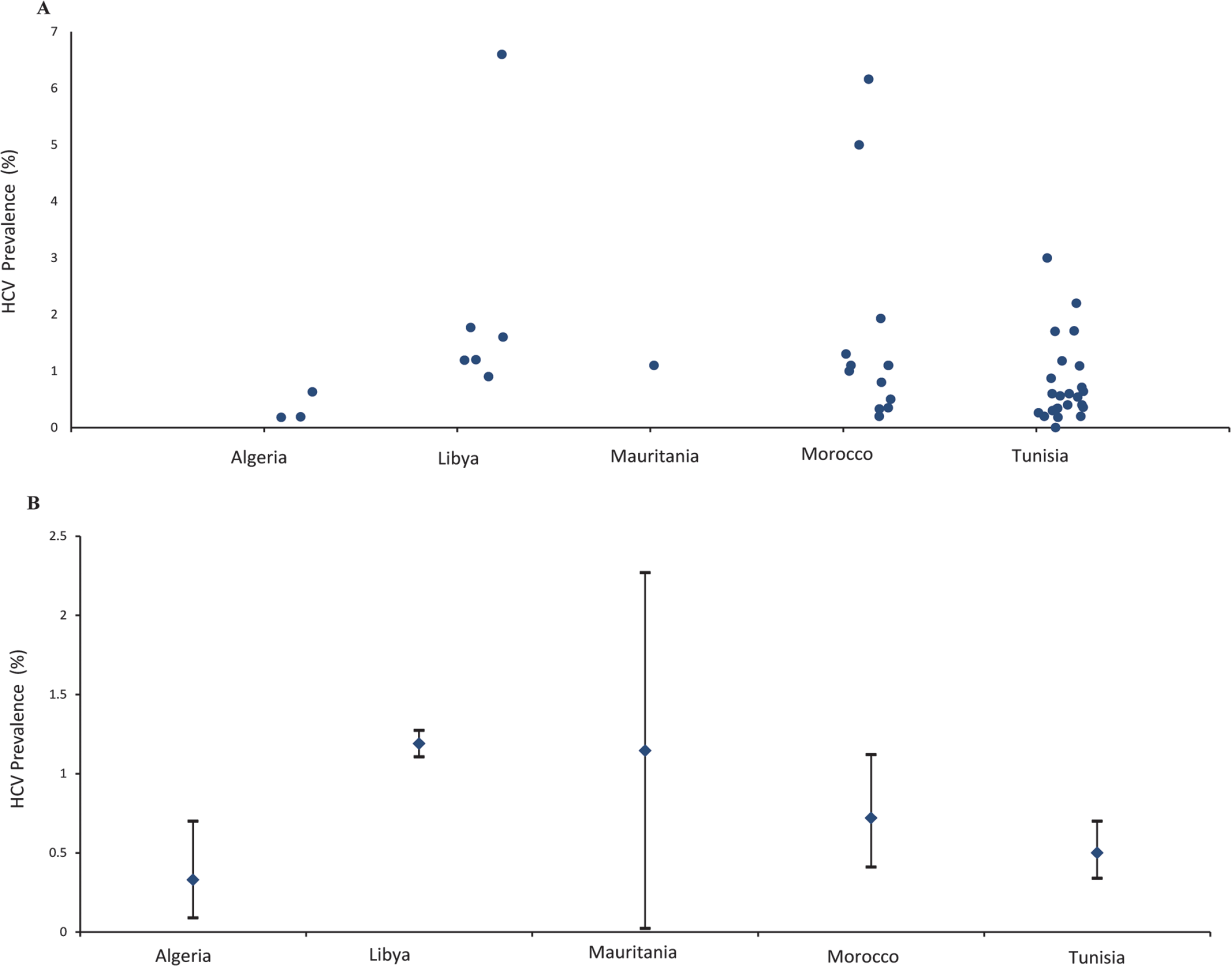
Hepatitis C virus (HCV) prevalence in the general population of the Maghreb countries. **A.** Available HCV prevalence measures among the general population as abstracted from studies included in the systematic review. **B.** Estimated HCV prevalence at the national level in each of the Maghreb countries.

All individual study estimates for HCV prevalence in the general population are presented in Fig. 2A. Using these measures, we estimated the national population-level HCV prevalence for each of the five countries (Fig. 2B). In Libya, since there was a population-based survey, we reported HCV prevalence as measured in this survey, of 1.2% (95% CI: 1.1–1.3) [101]. In Mauritania, since there was only one general population study conducted, we reported HCV prevalence in this study, of 1.1% (95% CI: 0–2.3), as an estimate of the national population-level HCV prevalence [34].

**Fig 3:**
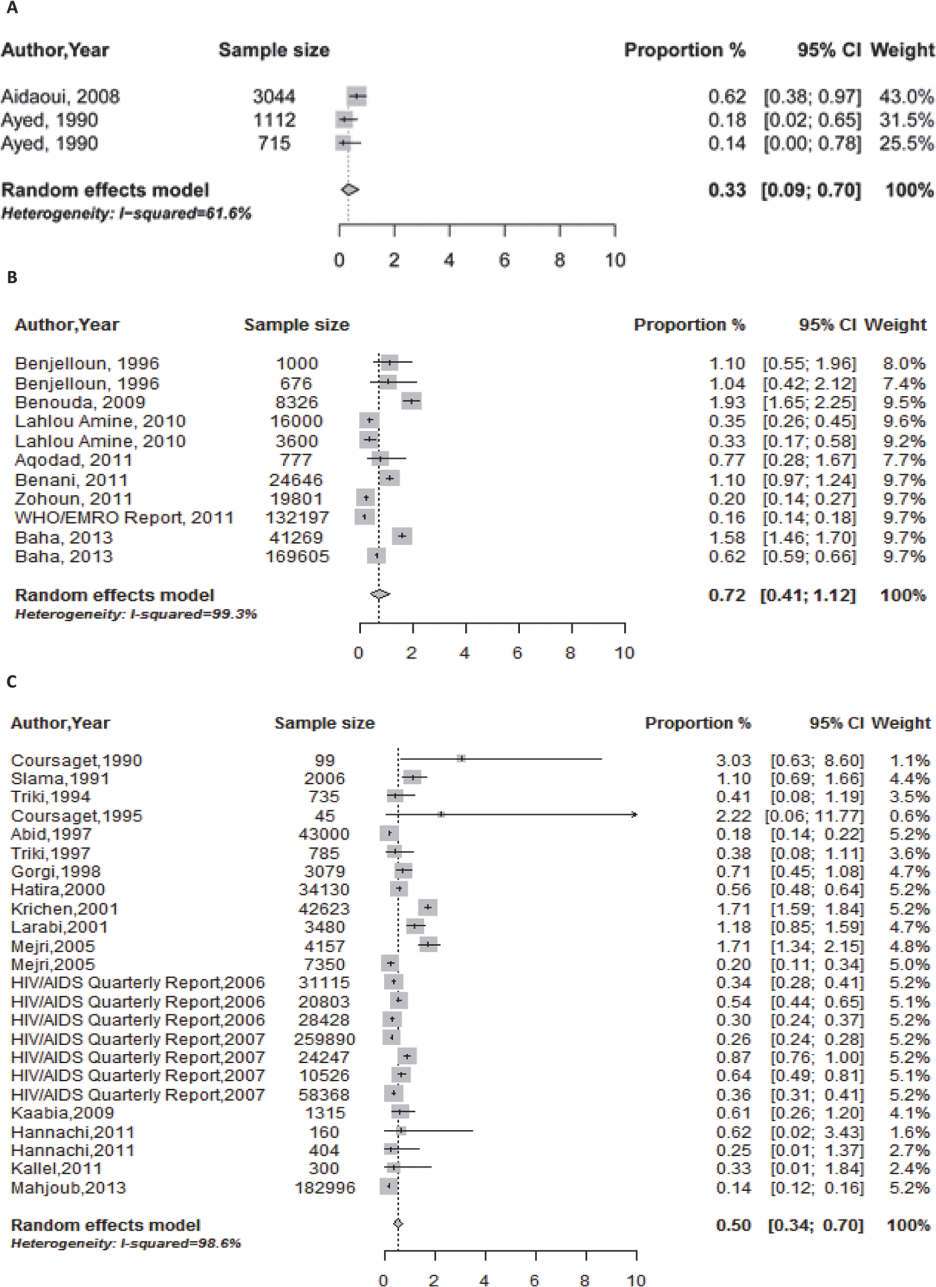
Pooled summary estimates of hepatitis C virus (HCV) prevalence among general population groups in A. Algeria; B. Morocco; C. Tunisia.

Fig. 3A, 3B, and 3C provide forest plots depicting the specific study estimates as well as the meta-analysis estimates for Algeria, Morocco and Tunisia. The pooled estimate for the national population-level HCV prevalence in Algeria was 0.3% (95% CI 0.1–0.7), I^2^ = 61% (95% CI 0.0–87.7, p = 0.10). The pooled estimate in Morocco was 0.7% (95% CI 0.4–1.1), I^2^ = 99% (95% CI 99.0–99.3, p < 0.001). The pooled estimate in Tunisia was 0.5% (95% CI 0.3–0.7), I^2^ = 99% (95% CI 98.8–99.1, p < 0.001).

## Discussion

We provided a comprehensive systematic review and synthesis of HCV prevalence and incidence in the Maghreb countries using a well-defined and thorough methodology and following the PRISMA guidelines. We highlighted the key features of HCV epidemiology in this region and, importantly, estimated the national population-level HCV prevalence in each of the Maghreb countries. With the recent remarkable successes in HCV treatment with direct-acting antivirals [102–105], our results take on additional importance as they provide the evidence base necessary for planning of health services, articulation of HCV policy guidelines, and implementation of HCV programming.

Our findings highlight that overall HCV prevalence in the Maghreb countries is at a level of about 1%, comparable to that observed in developed countries [1, 106]. This prevalence level is at odds with that observed in Egypt, the immediate eastern neighbor to the Maghreb, where HCV prevalence is estimated at 15% of the adult population [3, 4]. Additionally, the genotypes most reported in this region vary from that in Egypt (>95% of infections in Egypt belong to genotype 4) [99]. While no genotype information is available for Mauritania, reports from Algeria, Libya, Morocco, and Tunisia suggest genotype 1 as most common [37, 60, 99, 100, 107], in similar pattern to most countries globally [99, 108]. This finding highlights the uniqueness of the Egyptian epidemic and that the major drivers of this epidemic do not appear to be present in North Africa apart from Egypt. It further suggests that the infection transmission networks across the countries of North Africa may not have a sizable overlap.

Nonetheless, there is some evidence that the Egyptian epidemic may have affected HCV patterns in Libya, the neighboring country to the west of Egypt, and possibly Tunisia, the second nearest neighbor to Egypt of the Maghreb countries. HCV genotype 4 appears to account for more than one-third of HCV infections in Libya and one-quarter of infections in Tunisia [22, 99]. This suggests possible cross-over from Egypt into Libya, and then possibly into Tunisia. As many Egyptian migrant workers seek better employment opportunities in Libya, cross border migration may have facilitated avenues for the circulation of HCV from Egypt into Libya. It is possible too that a large fraction of genotype 4 cases in Libya could be reflecting Egyptian migrant workers rather than Libyan nationals. Since genotype 4 is also prevalent in Central Africa [99], it is possible that genotype 4 may have circulated into Libya and Tunisia through links to this part of Africa [109]. Of note that genotype 4 appears to have very limited presence in Algeria and Morocco [99].

Further epidemiological studies are needed to clarify the overlapping chains of transmission across North Africa. Despite the existence of at least some circulation of HCV from Egypt into Libya, the national HCV prevalence in Libya is comparable, though slightly higher, to the rest of the Maghreb countries, and an order of magnitude smaller than that in Egypt [4]. This further highlights the uniqueness of the Egyptian epidemic.

Overall, relatively high HCV prevalence levels were documented among individuals in high risk groups. Dialysis patients and transplant recipients, for example, have rates ranging from 16% [60] to as high as 76% [36]. Studies on HCV incidence among dialysis patients reported high HCV incidence rates of 3 [68] and 10 [37] per 100 person-years. Additionally, duration of dialysis, number of transfusions, and surgical and dental procedures were consistently cited as major risk factors across studies. HCV prevalence levels remained high in these populations, regardless of year of study, indicating possible ongoing transmission in healthcare settings. These findings suggest that a substantial fraction of HCV infections reflect healthcare-related exposures.

Injecting drug use (IDU) is the largest contributor to current HCV incidence in developed countries [110]. However, our findings indicate that this is not necessarily the case in the Maghreb countries. The high HCV prevalence and incidence levels among high risk populations exposed at healthcare facilities (Table 1), such as dialysis patients, suggest that IDU may not be the dominant contributor. Moreover, the prevalence of IDU in the Maghreb countries is lower than global levels, and smaller than that in the eastern part of MENA [111]. The prevalence of IDU was estimated at 0.22% in Algeria, 0.14% in Libya, 0.10% in Morocco, and 0.21% in Tunisia [111]. These rather low levels, in addition to an overall HCV prevalence of about 50% among PWID in MENA [111], suggest that IDU can explain only a minority of prevalent infections. However, given the severity of the HCV and HIV epidemics in Libya among PWID [112], it is possible that IDU may play a proportionally larger role in Libya than the rest of the Maghreb countries.

Our results further suggest that other exposures to HCV, in populations at intermediate risk, appear to be present. Much of these seem to be also related to healthcare, as can be seen by the higher HCV prevalence among diabetics, health care workers, and hospitalized populations (Table 1). Prisons appear to be a setting where a significant level of HCV exposure is found (Table 1), probably because of IDU prior to or after incarceration, but also possibly due to tattooing and sharing of utensils in prisons [7, 111]. Some exposures also seem to be related to certain professions or practices, such as among traditional barbers where somewhat considerable HCV prevalence is found (Table 1). It appears also that there is a rather significant HCV prevalence among sexual high risk populations, such as male and female sex workers and STD patients (Table 1). It is not clear though whether these prevalence levels reflect HCV sexual transmission or probably just higher IDU levels among these populations [111, 113].

There is a hint of geographic variability in HCV prevalence between different regions within the same country in both Libya and Tunisia. According to the Libyan population-based survey, HCV prevalence ranged from as low as 0.6% in Misrata to as high as 2.2% in Fezzan [13]. Similarly in Tunisia, HCV prevalence was estimated at 0.2% in the south and 1.7% in the northwest [82]. Such localized higher HCV prevalence levels have been observed in other settings globally such as in Japan and Taiwan [114–116]. They may suggest specific risk factors for HCV exposure at these localities that need to be investigated and identified. It is not clear for example whether such regional variations may reflect variations in infection control measures across the country, say in central health care facilities versus local facilities, or differences in the prevalence of IDU, or possibly the existence of localized traditional medicine practices that may expose individuals to HCV infection such as cautery and skin scarifications. Such factors that can drive geographically different infection transmission patterns appear to be present in MENA [7, 117]. Mapping of HCV exposure spatially, as has been done recently in Egypt [118], can shed light on such local risk factors and may point to wider issues of infection control that are beyond HCV concerns.

Our study provided country-specific estimates of HCV prevalence in the Maghreb using systematic review and meta-analysis methodology, a distinct approach from that used by the Global Burden of Disease (GBD) study [1]. Our approach is empirically focused whereby data on HCV prevalence are compiled through a systematic methodology and then pooled through formal statistical methods to yield estimates. The GBD approach utilizes mathematical modeling whereby systematically-compiled input data are used to parametrize complex models to generate the estimates. While our country-specific estimates are produced using data only from the respective country, GBD models are parametrized by data from different countries. While the overarching aim of our approach is to delineate the epidemiology of the infection, the GBD’s approach aims to reach estimates for the disease burden resulting from complications of this infection. These two approaches therefore should be seen as complementary approaches, each of which has its strengths and limitations, and each of which is informing the other.

Among the limitations of our study are the variability of the number studies across countries and the low number of studies from Algeria and Mauritania (only one study was identified in Mauritania). The general population studies included in the meta-analyses may not have been representative of the population at large. Nearly all studies were on convenient samples and there was only one study on a nationally representative and probability-based sample (in Libya; Table 1). Many of the studies, particularly the large ones, were among blood donors or pregnant women. HCV prevalence in these populations may underestimate HCV prevalence in the whole population; there could be selection towards lower risk among blood donors, and women tend to have lower HCV prevalence than men.

Our meta-analyses highlighted that there is substantial heterogeneity among the studies conducted in general population groups. This is not surprising considering the differences between studies in terms of the specific general population studied, sampling methodology and participant recruitment, age-group representation in the sample, year of study, location and geographic sub-region of study, and assay used. However, due to the small number of observations for each country, we were unable to conduct a meta-regression analysis to identify potential sources of variation to explain the observed heterogeneity for each country.

We classified the populations into high risk, intermediate risk, and general population (low risk) groups by convention in HCV epidemiology literature. However, there is no established existing classification of risk for some populations, and the information available in some studies was not sufficient to determine the level of risk. In these situations the level of risk was determined based on our best judgment of the risk of exposure to HCV infection in this population. For example, HIV infected patients who have acquired HIV likely through a parenteral mode of transmission, were classified as a high risk population, whereas HIV infected persons who have acquired HIV likely though a sexual mode of transmission, were classified as an intermediate risk population. Furthermore, clinical populations for which the risk of exposure was uncertain were classified into an independent category as *special clinical populations*.

Another limitation in our study is that there was variability in the diagnostic assays used across studies. Earlier studies typically reported the use of 1^st^ and 2^nd^ generation ELISA tests, which lack the sensitivity and specificity of the 3^rd^ generation ELISA tests. Such variability in assays may impact the representativeness of earlier studies. Lastly, only two studies in this region provided empirical measures of HCV incidence, and these were fairly dated.

## Conclusion

HCV prevalence in the Maghreb region of MENA is comparable to that in developed countries of about 1%. Yet, the evidence synthesized here suggests ongoing HCV transmission through specific high-risk healthcare exposures such as dialysis and blood transfusions. IDU is also a major, though probably not dominant, contributor to HCV transmission in these countries. Other exposures, though at much reduced risk, appear to be related to intermediate-risk healthcare settings or procedures and specific professions or community practices. Further research is needed to draw a more thorough and complete understanding of HCV epidemiology in this part of MENA, especially so in the countries where a limited number of studies have been conducted (Algeria and Mauritania). Our findings suggest the need for a targeted approach to control HCV transmission by focusing the response on the settings of exposure. The findings also provide the evidence base necessary to inform planning of health service provision, articulation of HCV policy guidelines, and implementation of HCV programming to reduce HCV transmission and decrease the burden of its associated diseases.

## Supporting Information

S1 Table PRISMA checklist. (DOCX)

S1 Box Search criteria. (DOCX)
