# Supplementary material for: The Epidemiology of Hepatitis C Virus in the Maghreb Region: Systematic Review and Meta-Analyses
Source: PLoS One. 2015 Mar 24;10(3):e0121873. doi: 10.1371/journal.pone.0121873 (PMC4372394; doi:10.1371/journal.pone.0121873)
Supplement: S1 Box — (DOCX) [file pone.0121873.s002.docx]

**Supporting Information S1 Box**

**Search Criteria**

**PubMed**

("Hepatitis C"[Mesh] OR "Hepatitis C Antibodies"[Mesh] OR "Hepatitis C Antigens"[Mesh] OR “Hepacivirus”[Mesh] OR “Hepatitis C, chronic/epidemiology”[Mesh] OR “Hepatitis C, chronic/etiology”[Mesh] OR “Hepatitis C, chronic/transmission”[Mesh] OR “Hepatitis C, chronic/virology”[Mesh] OR “Hepatitis C”[Text] OR “HCV”[Text]) AND ((“Africa,Northern” [Mesh] or “Algeria” [Mesh] or “Libya” [Mesh] or “Morocco” [Mesh] or “Tunisia” [Mesh] or “Mauritania” [Mesh] or “Algeria” [Text] or “Libya” [Text] or “Morocco” [Text] or “Tunisia” [Text] or “Mauritania” [Text] or “Algeria*” [Text] or “Libya*” [Text] or “Moroccan*” [Text] or “Tunis*” [Text] or “Mauritania*” [Text] or “North Africa” [Text] or “North-Africa” [Text] or (“Africa” [Text] AND “Northern” [Text]) or “Northern Africa” [Text] or “Maghreb” [Text] or “Maghrib”[Text]))

**Embase**

(exp North Africa/ or North Africa.mp. or Maghreb.mp. or Algeria*.mp. or exp Algeria/ or Libya*.mp. or exp Libya/ or Morocc*.mp. or Morocco*.mp. or Moroccan*.mp. or exp Morocco/ or Tunisia*.mp. or exp Tunisia/ or Mauritania*.mp. or exp Mauritania/) AND (exp hepatitis C/ or exp Hepatitis C virus/ or hepatitis C.mp. or HCV.mp. or hepacivirus.mp.)
